# Combining ^99m^Tc-GSA single-photon emission-computed tomography and Gd-EOB-DTPA-enhanced magnetic resonance imaging for staging liver fibrosis

**DOI:** 10.1097/MD.0000000000032975

**Published:** 2023-02-17

**Authors:** Ryotaro Tokorodani, Toshiaki Kume, Hiromitu Daisaki, Naoya Hayashi, Hitomi Iwasa, Takuji Yamagami

**Affiliations:** a Division of Radiology, Department of Medical Technology, Kochi Medical School Hospital, Nankoku, Japan; b Department of Radiological Technology, Kochi Health Sciences Center, Kochi, Japan; c Department of Gunma Prefectural College of Health Sciences, Maebashi, Japan; d Department of Diagnostic and Interventional Radiology, Kochi Medical School, Kochi University, Nankoku, Japan.

**Keywords:** ^99m^Tc-GSA, CEI, Gd-EOB-DTPA, liver fibrosis, SUV

## Abstract

Preoperative assessment of the degree of liver fibrosis is important to determine treatment strategies. In this study, galactosyl human serum albumin single-photon emission-computed tomography and ethoxybenzyl (EOB) contrast-enhanced magnetic resonance imaging (MRI) were used to assess the changes in hepatocyte function after liver fibrosis, and the standardized uptake value (SUV) was combined with gadolinium EOB-diethylenetriaminepentaacetic acid to evaluate its added value for liver fibrosis staging.

A total of 484 patients diagnosed with hepatocellular carcinoma who underwent liver resection between January 2010 and August 2018 were included. Resected liver specimens were classified based on pathological findings into nonfibrotic and fibrotic groups (stratified according to the Ludwig scale). Galactosyl human serum albumin-single-photon emission-computed tomography and EOB contrast-enhanced MRI examinations were performed, and the mean SUVs (SUV_mean_) and contrast enhancement indices (CEIs) were obtained. The diagnostic value of the acquired SUV and CEIs for fibrosis was assessed by calculating the area under the receiver operating characteristic curve (AUC).

In the receiver operating characteristic analysis, SUV + CEI showed the highest AUC in both fibrosis groups. In particular, in the comparison between fibrosis groups, SUV + CEI showed significantly higher AUCs than SUV and CEI alone in discriminating between fibrosis (F3 and 4) and no or mild fibrosis (F0 and 2) (AUC: 0.879, vs SUV [*P* = 0.008], vs. CEI [*P* = 0.023]), suggesting that the combination of SUV + CEI has greater diagnostic performance than the individual indices.

Combining the SUV and CEI provides high accuracy for grading liver fibrosis, especially in differentiating between grades F0 and 2 and F3–4. SUV and gadolinium EOB-diethylenetriaminepentaacetic acid-enhanced MRI can be noninvasive diagnostic methods to guide the selection of clinical treatment options for patients with liver diseases.

## 1. Introduction

The degree of fibrosis is an important indicator of liver function, as organ damage leads to fibrotic changes in the liver parenchyma. Cirrhosis represents the most advanced stage of liver fibrosis and is associated with a potential risk of malnutrition, coagulopathy, fatal liver failure, and posing restrictions in performing invasive therapeutic procedures.^[1–4]^ Therefore, assessing the degree of fibrosis based on the patient preoperative assessment is important for determining treatment strategies. Liver biopsy remains the reference standard; however, it is invasive,^[5,6]^ carries the risk of hemorrhagic complications, is subject to sampling errors. In addition, sampling is difficult because of the small and hard liver in advanced disease, interobserver variations exist.^[7,8]^ Therefore, noninvasive assessment of fibrosis by imaging, which is repeatable and less invasive, is an important area of study.^[9–13]^

Ultrasound (US) elastography is a noninvasive and reliable method of assessing liver fibrosis.^[14–17]^ However, US examination depends on the examiner skill, the narrow field-of view makes it impossible to assess the entire liver. Liver stiffness, mainly caused by fibrosis, can be influenced by patient-dependent factors such as liver inflammation, congestion and biliary obstruction.^[18–20]^ Hence, elastographic results should be interpreted in the full clinical context of patient-related factors.

Similarly, magnetic resonance imaging (MRI) can be used to measure liver stiffness by elastography. MR elastography uses mechanical waves to measure tissue elasticity to stage liver fibrosis, with significantly high sensitivity and specificity.^[21,22]^ However, MR elastography may not be available in many institutions because of its complex mechanical setup, dedicated MRI sequence, and lengthy image acquisition.

Gadolinium ethoxybenzyl-diethylenetriaminepentaacetic acid (Gd-EOB-DTPA) is a hepatocyte-specific MRI contrast agent.^[23]^ It acts as an extracellular contrast agent in the early phase after intravenous injection, and it is subsequently taken up by the hepatocytes. It enhances the detection and diagnosis of hepatocellular carcinoma and is considered a functional contrast medium.^[24,25]^ Studies on the hepatocyte-phase-contrast enhancement index (CEI) in the staging of liver fibrosis in recent years have reported that the CEI is more reliable than diffusion-weighted MRI and hematological and clinical parameters for staging liver fibrosis.^[26]^ The assessment of fibrosis using the CEI is highly reproducible and allows easy assessment of the whole liver.^[27,28]^

Similarly, the assessment of fibrosis using Tc-99m-diethylenetriamine-penta-acetic acid-galactosyl human serum albumin (^99m^Tc-GSA) is a reproducible and easy method for assessing the whole liver.^[13,29–31]^ Recent studies have reported that standardized uptake value (SUV) quantification is possible in single-photon emission-computed tomography (SPECT-CT) with high accuracy of attenuation, scatter, and resolution correction.^[32]^ SUV assessment of galactosyl human serum albumin (GSA) accumulation has also been reported.^[29]^ SUVs represent the concentration of radiopharmaceutical substances in target organs and are easily obtained and highly reproducible indicators.^[33]^

GSA-SPECT-CT and ethoxybenzyl (EOB) contrast-enhanced MRI scans enable the assessment of accumulation in images, highly reproducible, and can be easily integrated into routine imaging protocols. Both tests were performed to assess liver function before hepatectomy and to assess the degree of fibrosis in the background liver without additional burden on the patient. We used GSA-SPECT-CT and EOB contrast-enhanced MRI to assess the changes in hepatocyte function after liver fibrosis, further combined SUV with Gd-EOB-DTPA to evaluate the benefit for liver fibrosis staging.

## 2. Methods

### 2.1. Patients

This study included 484 patients diagnosed with hepatocellular carcinoma, who underwent hepatic resection consecutively between January 2010 and August 2018. Patients who met the following criteria were included in the analysis: ^99m^Tc-GSA-SPECT-CT fusion study performed within 1 week prior to surgery; EOB-MRI study conducted within 1 month prior to surgery; no history of liver resection; and pathologists were able to assess fibrosis of the liver in the nonneoplastic area. Of the 484 patients, 254 were excluded because either GSA or MRI was not performed or the period during which it was performed was outside the period specified in the inclusion criteria. Of the remaining 230 patients, 56 were excluded because they had previously undergone hepatectomy, which may not allow an accurate region-of interest (ROI) to be established, and of the remaining 174 patients, 16were excluded because a histological diagnosis could not be made by a pathologist. Finally, a total of 158 patients (121 males and 37 females; mean age, 70.7 [range: 38–81] years) were included in the analysis. We obtained ethics review board certification from our institution. This study was conducted in accordance with the ethical standards of the 1964 Declaration of Helsinki and its later amendments or comparable ethical standards.

### 2.2. ^99m^Tc-GSA-SPECT-CT fusion imaging

At our institution, ^99m^Tc-GSA-SPECT-CT fusion imaging is routinely used in hepatectomy candidates to evaluate liver function. All patients underwent examination using a Symbia T6 scanner (Siemens, Munich, Germany), which combines variable-angle dual-detector SPECT with 6-slice CT for rapid and accurate attenuation correction, precise localization, and seamless transition from SPECT examination to CT examination. SPECT and CT images can be obtained in a single examination, without repositioning.

The procedure for the investigation was as follows. After overnight fasting, patients were placed in a supine position. Cardiac and respiratory synchronization were not used in this modality. Instead, to minimize the possibility of artifacts due to cardiac pulsation and respiratory motion, the patients were encouraged to rest and breathe slowly before image acquisition. ^99m^Tc-diethylenetriamine-penta-acetic acid-GSA (Nihon Medi-Physics, Tokyo, Japan) (185 MBq/3 mg) was injected into the antecubital vein. SPECT data acquisition (60 steps of 20 seconds/step, 360°, 128 × 128 matrix) was started 20 minutes after injection using a low-energy, high-resolution collimator; the entire study duration was approximately 30 minutes. The reconstruction algorithm for SPECT was 3-dimensional ordered subset expectation maximization with attenuation and scatter corrections. Following the SPECT examination, nonenhanced CT images were obtained under the standard conditions of 130 kV, 345 mA, 12-mm table feed per rotation, 0.6-second gantry rotation time, 0.6-mm collimation, and 1-mm reconstruction. CT images were reconstructed using a standard algorithm with a 166-cm field-of-view of the target sites. The SPECT and CT images were fused automatically using the embedded Siemens common platform software (Syngo MI Workplace, Siemens Healthcare, Germany). SPECT slice data were retrieved through digital imaging and communication in medicine, and SPECT slices were converted to a CT-like data volume for the fusion of the SPECT and CT images.

### 2.3. EOB-MRI imaging

EOB-MRI has been routinely applied to evaluate liver function in hepatectomy candidates at our institution. MRI was performed using Signa HDx 1.5T (GE Healthcare, Milwaukee, WI) with an 8-channel phased-array coil. The images were acquired in the transverse plane with a section thickness of 5-mm and a 2.5-mm overlap (i.e., 2.5-mm interval). Repetition time/echo time was 4.1/1.9 ms, flip angle was 12°, number of signals acquired was 1, field-of view was 360 × 288 mm, matrix was 288 × 160, and scan time was 12 seconds. Gd-EOB-DTPA (Primovist, Bayer Schering Pharma, Berlin, Germany) (0.1 mg/kg body weight) was administered intravenously as a bolus dose at a rate of 3 mL/s through an intravenous cubital line that was flushed with 20 mL saline using a power injector. Imaging was performed before contrast and 30 seconds, 60 seconds, 180 seconds, and 20 minutes after the start of contrast injection, and the image at 20 minutes was used as the hepatocellular phase.

### 2.4. Calculation of SUV_mean_

The accumulation of ^99m^Tc-GSA in the liver was evaluated using the SUV.^[29,32]^ Decay correction was applied to control the fluctuation at the start time of the acquisition. The SUV value was normalized by the liver volume, which was calculated automatically using workstation VINCENT (Fujifilm, Tokyo, Japan).^[34]^

SUV was calculated using the following formula:


$$\begin{array}{l} SUV_{}=\;\frac{Radioactivity\;of\;liver\;VOI\;\left(Bq/mL\right)}{\begin{array}{c} Dose\;at\;the\;start\;of\;scan\;(Bq)\\ /Liver\;volume\;\left(mL\right)\times 10 \end{array}} \end{array}$$

**Figure 1.**
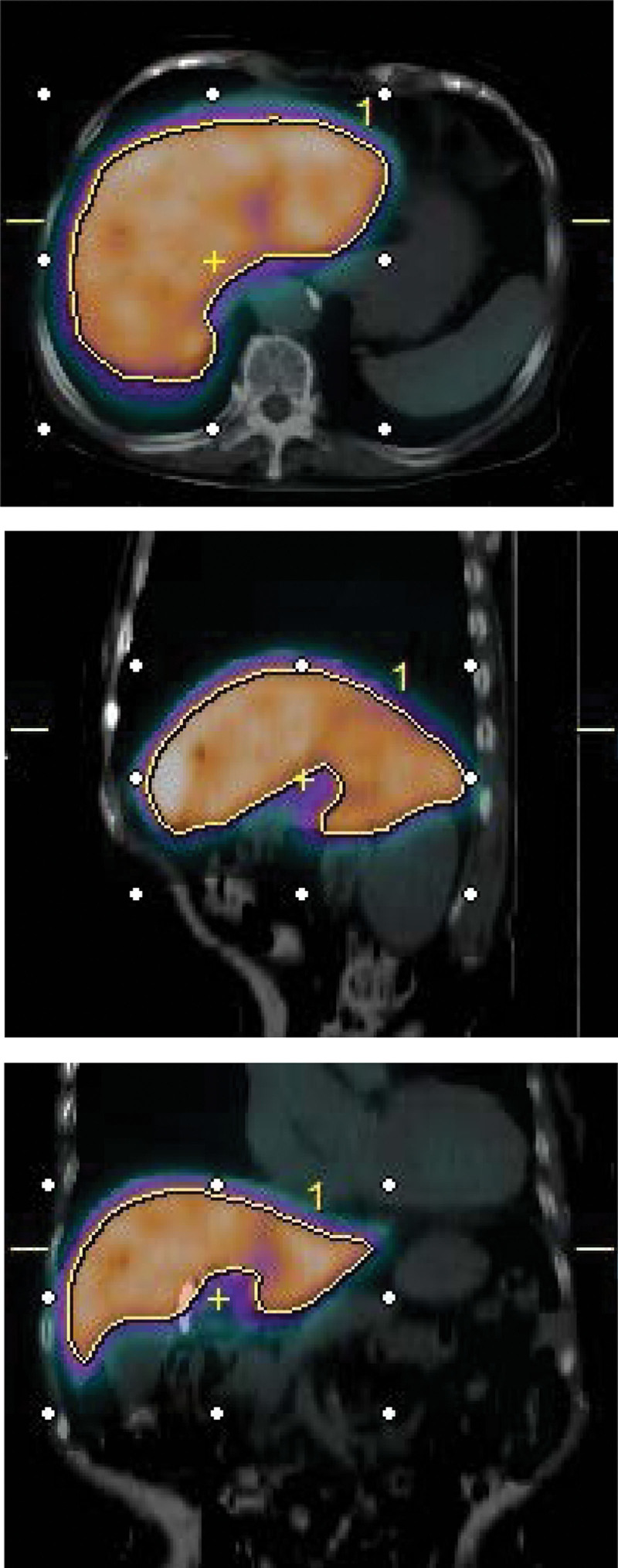
To measure the mean standardized uptake value (SUV_mean_), a region-of interest (ROI) was established over the entire liver on single-photon emission-computed tomography (SPECT-CT) fusion image. All galactosyl human serum albumin (GSA) SPECT images were acquired 20 minutes after intravenous Tc-99m-diethylenetriamine-penta-acetic acid (^99m^Tc)-GSA injection. ROI setting was performed using GI-BONE; GI-BONE is an application for SUV calculation that automatically sets the threshold value for ROI. The threshold value was set to SUV 3. ROI = region-of interest, SPECT-CT = single-photon emission-computed tomography. SUV = standardized uptake value.

Setting the volume of interest (VOI) at the site of ^99m^Tc-GSA accumulation in the liver is necessary to calculate the SUV; therefore, we used the commercially available software GI-BONE (AZE Co., Ltd., Tokyo, Japan), which sets the VOI automatically (Fig. 1). The VOI was placed to contain the entire liver. The software automatically detected the region-of voxels with an SUV > 3, and the mean SUV (SUV_mean_) in the designated region was calculated. Furthermore, ^99m^Tc-GSA is taken up only in the liver, not the whole body; therefore, the liver volume was utilized to normalize the radioactivity in this study.

### 2.5. Calculation of CEI

**Figure 2.**
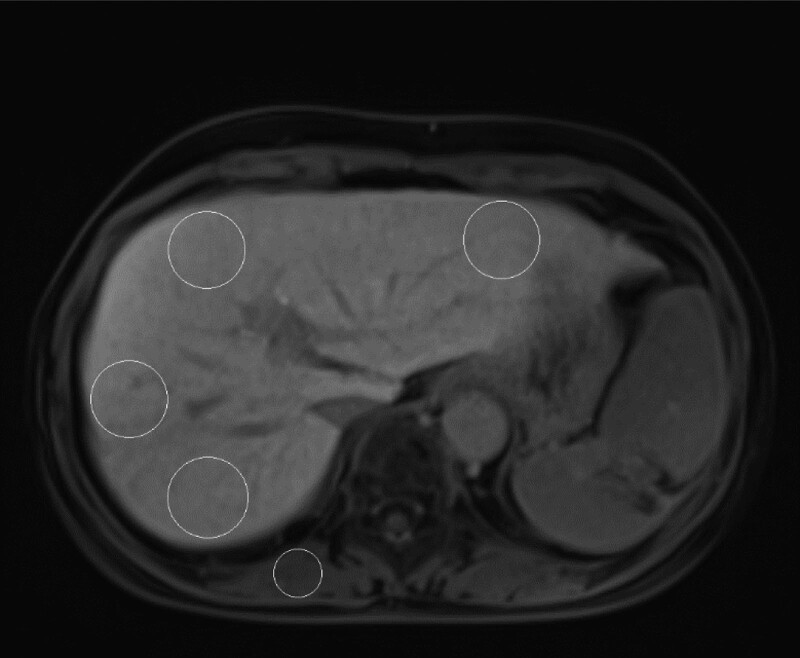
To measure the contrast enhancement index (CEI), regions of interest (ROIs) were set up on precontrast images and gadolinium ethoxybenzyl-diethylenetriaminepentaacetic acid (Gd-EOB-DTPA)-weighted liver phase images. ROIs were set up in 4 regions of the paraspinal muscle and liver parenchyma and averaged; ROI sizes were 4 cm^2^ for the liver and 1–2 cm^2^ for the paraspinal muscle. To minimize differences in signal intensity due to near-field effects, the ROIs for the liver and paraspinal muscles were selected at a fixed distance from the abdominal wall. Gd-EOB-DTPA = Gadolinium ethoxybenzyl-diethylenetriaminepentaacetic acid, ROI = region-of interest.

Quantitative measurements were conducted on nonenhanced and gadoxetate disodium-enhanced hepatocyte-phase images using operator-defined ROI (4 cm^2^) measurements of the mean signal intensity (SI) values of the liver and paraspinal muscle measured as follows. The SI values of the liver were measured in areas devoid of focal changes in the SI, large vessels, or prominent artifacts. To minimize the differences in SI due to the near-field effect, ROIs in the liver and paraspinal muscles were located such that the distances from the abdominal walls were consistent (Fig. 2). Measurements were acquired by 2 radiologists with sufficient experience (9 and 18 years of experience) in ROI setting and MRI image processing.

The liver SI ratio was calculated as the ratio of hepatic SI to paraspinal muscle SI separately on nonenhanced images (liver-to-muscle SI ratio on nonenhanced images [SI pre]) and the ratio of hepatic SI to paraspinal muscle SI on hepatocyte-phase images (liver-to-muscle SI ratio on hepatocyte-phase images [SI post]). CEI was calculated as follows: $CEI\;=\;\frac{SI\;post}{SI\;pre}$

SYNAPSE VINCENT (Fujifilm) was used for the ROI setting.

### 2.6. Degree of fibrosis

The degree of fibrosis was pathologically diagnosed in the liver parenchyma, apart from the liver tumor, in each resected specimen. The Ludwig scale was used to stratify the grade of fibrosis: F1 (no-fibrosis or fibrosis confined to enlarged portal tracts), F2 (periportal fibrosis or portal-to-portal septa but intact architecture), F3 (septal fibrosis with architectural distortion), and F4 (probable or definite cirrhosis). The degree of fibrosis was assessed by 2 pathologists who were blinded to patient characteristics.^[35]^

### 2.7. Statistical analyses

The 1-sample Kolmogorov–Smirnov test was used to test the normal distribution of the quantitative variables. A normality test could not verify the normality of variables. Therefore, nonparametric statistical tests were performed. The Kruskal–Wallis test was used for comparison, and the Mann–Whitney *U* test was performed for multiple-comparison test, which was corrected using the Holm method. The combination of SUV and CEI for assessing liver fibrosis was tested using multiple linear regression analysis; the fibrosis stage was considered a continuous variable, the SUV and CEI distributions were normalized by log transformation. The trend of continuous variables at each fibrosis stage were evaluated using the Jonckheere–Terpstra test. Differences between medians were considered statistically significant at *P* < .05. The diagnostic performance of SUV, CEI, and their combination for liver fibrosis was assessed using receiver operating characteristic (ROC) curves. In addition, the sensitivity; specificity; area under the receiver operating characteristic curve (AUC); positive predictive value; negative predictive value; and accuracy of SUV ratios, CEI values, and SUV + CEI were demonstrated. The differences between the AUCs were compared using the Delong test.^[36]^ These statistical analyses were performed using the Statistical Package for the Social Sciences version 24 for Windows (SPSS, IBM Corp., ver. 24, Armonk, NY) and MedCalc statistical software (MedCalc Software, Mariakerke, Belgium).

## 3. Results

### 3.1. Patient characteristics

Patient characteristics are shown in Table 1. The distribution of fibrosis stages among the 158 patients was F0, n = 15; F1, n = 17; F2, n = 40; F3, n = 42; and F4, n = 44. No adverse events occurred during the study.

**Table 1:** Clinical characteristics.

Variable	(n = 158)
Sex (male/female)	121/37
Age (range) (median)	70.7 ± 11.1 (39–89)
HCV antibody-positive, n	55
HBs antigen-positive, n	28
Alcohol abuse, n	22
Liver function data	
Platelet count (range), × 10^4^/μL	16.4 ± 5.6 (5.7–34.4)
AST (range), IU/L	53.7 ± 33.8 (16–192)
Total bilirubin (range), mg/dL	0.8 ± 0.6 (0.3–4.0)
Albumin (range), g/dL	4.0 ± 0.7 (1.5–4.9)
ICGR15 (range), %	16.1 ± 10.5 (0.6–57.3)
Fibrosis score (Ludwig scale)	
F0	15
F1	17
F2	40
F3	42
F4	44

AST = aspartate transaminase, ICGR15 = indocyanine green dye retention at 15 minutes, HBs = hepatitis B surface, HCV = hepatitis C virus.

### 3.2. Association between SUV and CEI measurements and liver fibrosis stages

**Figure 3.**
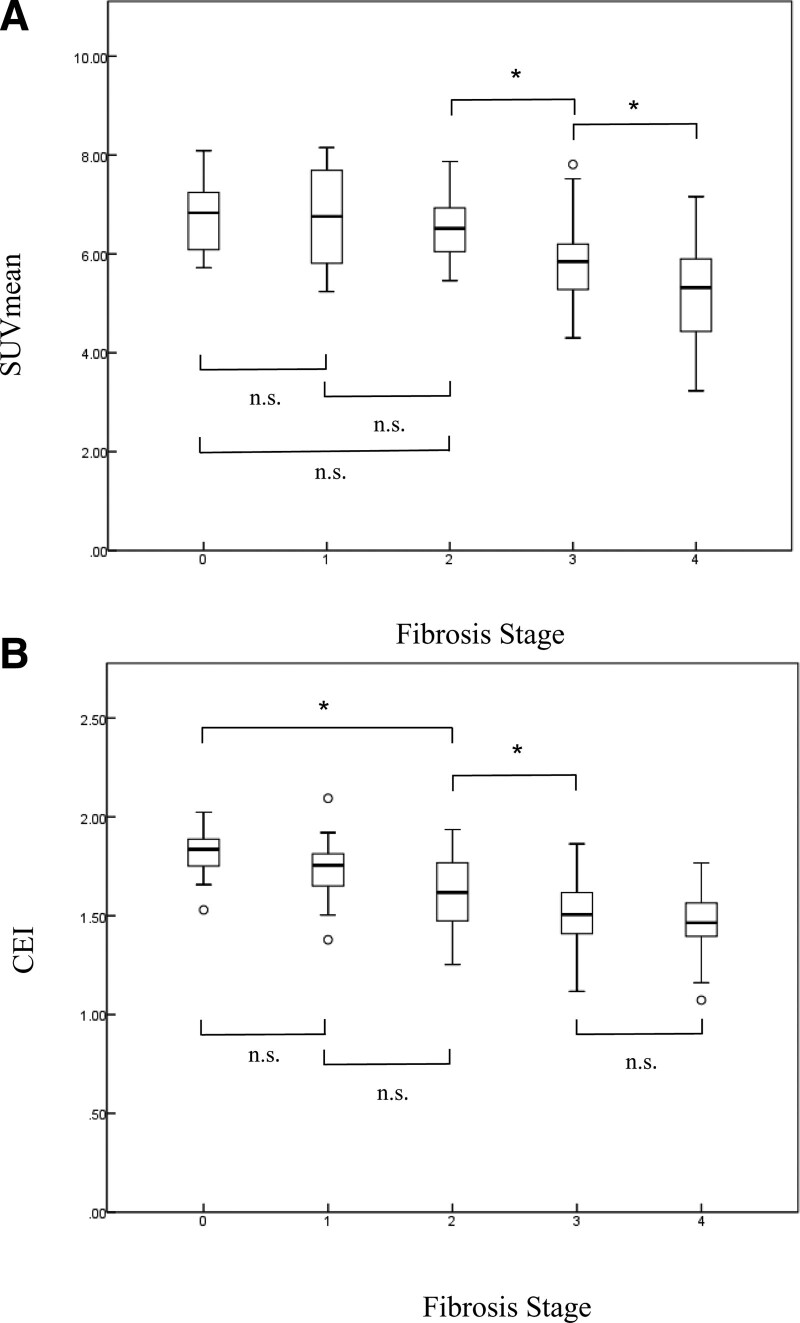
Box plots of the mean standardized uptake value (SUV_mean_) **(a**) and contrast enhancement index (CEI) **(b**) for each fibrosis stage. Boundary of boxes closest to zero = 25th percentile, line in boxes = median, boundary of boxes farthest from zero = 75th percentile, error bars = smallest and largest values in 1.5 box lengths of the 25th and 75th percentiles, ○ = outliers, and * = significant difference (*P* < .005). n.s.: not significant. SUV = standardized uptake value.

SUV and CEI measurements, according to fibrosis stage, are summarized in Table 2. Box plots of the SUV and CEI for each fibrosis stage are shown in Figure 3. SUV decreased consistently with increasing fibrosis stage (*P* < .005) but did not differ between stages F0 and F1, F0 and F2, and F1 and F2 (score of F0 vs F1, *P* = 1.000; F0 vs F2, *P* = 1.000; F1 vs F2, *P* = 1.000). Stages F2, F3, F3, and F4 were significantly different (F0 vs F2, *P* < .001; F2 vs F3, *P* = .043). CEI decreased consistently with increasing fibrosis stage (*P* < .005) but did not differ between stages F0 and F1, F1, F2, F3, and F4 (score of F0 vs F1, *P* = .284; F1 vs F2, *P* = .095; F3 vs F4, *P* = .441). The F0, F2, F2, and F3 stages showed significant differences (F0 vs F2, *P* < .001; F2 vs F3, *P* = .0313).

**Table 2:** SUV and CEI according to fibrosis stage.

Data	SUV	CEI
Fibrosis stage		
F0	5.72–8.09 (6.83 ± 0.74)	1.53–2.02 (1.84 ± 0.13)
F1	5.24–8.15 (6.76 ± 0.97)	1.38–2.09 (1.76 ± 0.16)
F2	5.46–7.87 (6.52 ± 0.58)	1.25–1.94 (1.62 ± 0.18)
F3	4.30–7.81 (5.85 ± 0.76)	1.12–1.86 (1.51 ± 0.18)
F4	3.23–7.16 (5.32 ± 0.96)	1.07–1.77 (1.46 ± 0.15)

Numbers in parentheses present the means ± standard deviation.

CEI = contrast enhancement index, SUV = standardized uptake value.

### 3.3. SUV and CEI ROC curve comparison

**Figure 4.**
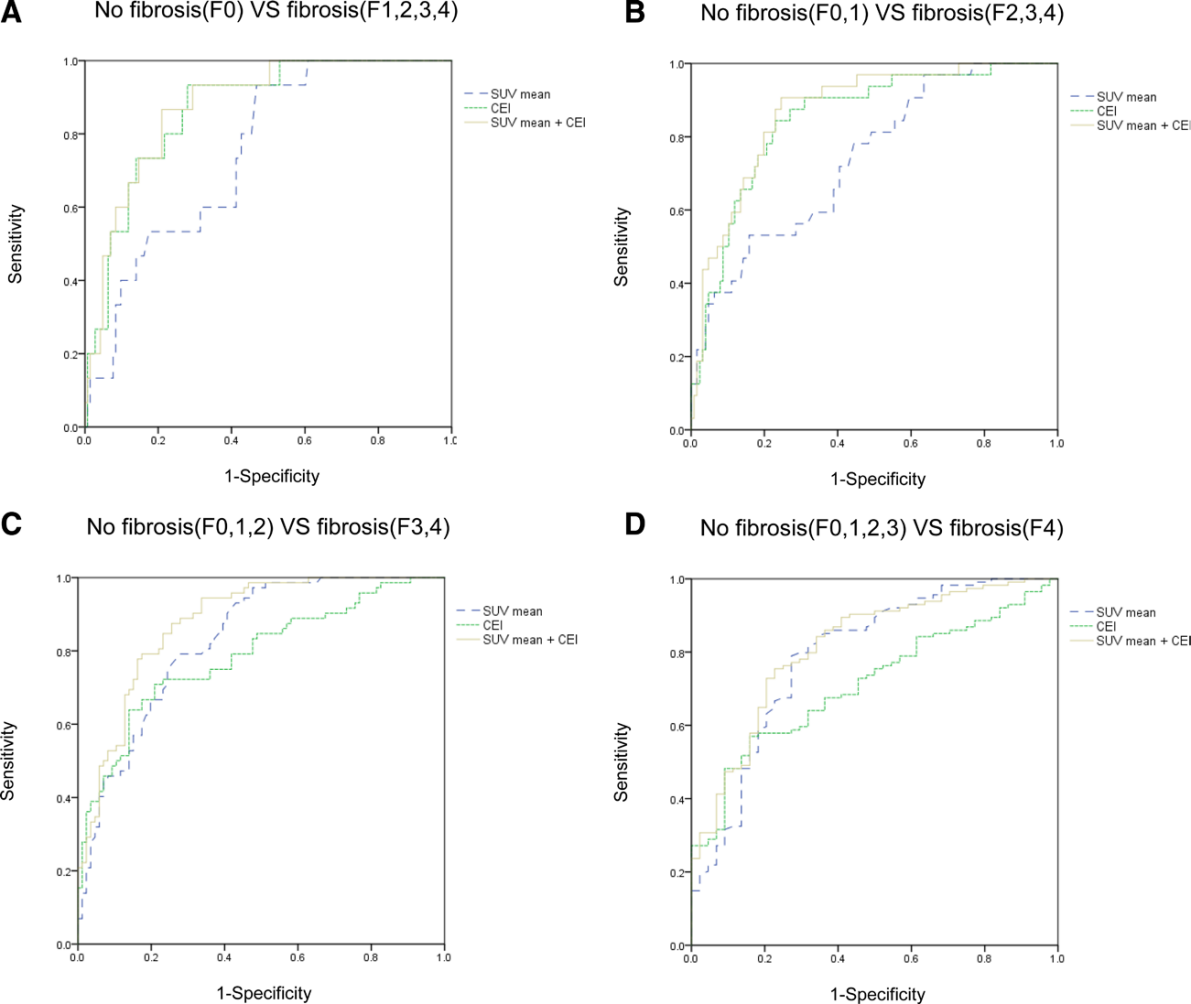
Comparison of the receiver operating characteristics curves of the mean standardized uptake value (SUV_mean_) and contrast enhancement index (CEI) for liver fibrosis diagnosis. (a) No-fibrosis (F0) versus fibrosis (F1–4); (b) no-fibrosis (F0, 1) versus fibrosis (F2–4); (c) no-fibrosis (F0–2) versus fibrosis (F3, 4); and (d) no-fibrosis (F0–3) versus fibrosis (F4). SUV = standardized uptake value.

ROC analysis was performed to assess the effectiveness of using the SUV and CEI to discriminate between the different stages of fibrosis (Fig. 4). Table 3 shows the AUCs and predictive values for SUV and CEI. CEI showed significantly higher AUCs in stages F0 and F1 to 4 (AUC: SUV vs CEI = 0.749 vs 0.868, *P* = .0419) and stages F0 to 1 and F2 to 4 (AUC: SUV vs CEI = 0.740 vs 0.849, *P* = .0471) than in SUV. In contrast, SUV had higher, although not significant, AUCs than CEI in stages F0 to 2 and F3 to 4 (AUC: SUV vs CEI = 0.830 vs 0.787, *P* = .3304), and in stages F0 to 3 and F4 (AUC: SUV vs CEI = 0.792 vs 0.713, *P* = .1463).

**Table 3:** Diagnostic performance of SUV_mean_, CEI, and SUV + CEI.

	AUC (95% CI)	Sn	Sp	PPV	NPV	Accuracy
SUV_mean_ fibrosis stage						
≥ F1	0.749 (0.639–0.859)	0.933	0.559	0.222	0.989	0.684
≥ F2	0.740 (0.647–0.834)	0.841	0.438	0.855	0.412	0.759
≥ F3	0.830 (0.768–0.892)	0.860	0.733	0.716	0.818	0.753
≥ F4	0.792 (0.709–0.875)	0.789	0.727	0.900	0.586	0.785
CEI fibrosis stage						
≥ F1	0.868 (0.788–0.948)	0.933	0.727	0.264	0.990	0.747
≥ F2	0.849 (0.777–0.922)	0.844	0.770	0.482	0.951	0.785
≥ F3	0.787 (0.714–0.859)	0.639	0.860	0.793	0.740	0.759
≥ F4	0.713 (0.631–0.795)	0.570	0.841	0.903	0.430	0.648
SUV_mean_ + CEI fibrosis stage						
≥ F1	0.877 (0.800–0.953)	0.867	0.790	0.302	0.983	0.797
≥ F2	0.870 (0.806–0.934)	0.906	0.746	0.475	0.969	0.778
≥ F3	0.879 (0.826–0.931)	0.875	0.773	0.733	0.875	0.797
≥ F4	0.815 (0.741–0.889)	0.754	0.750	0.887	0.541	0.753

Accuracy represents the ratios of patients who were classified appropriately (true positive + true negative).

AUC = area under the receiver operating characteristic curve, CEI = contrast enhancement index, CI = confidence interval, NPV = negative predictive value, PPV = positive predictive value, Sn = sensitivity, Sp = specificity, SUV = standardized uptake value.

Regarding F0 and F1 to 4 (AUC: 0.877 vs SUV [*P* = .009] vs CEI [*P* = .4197]), F0 to 1 and F2 to 4 (AUC: 0.870 vs SUV [*P* = .033] vs CEI [*P* = .1246]), F0 to 2 and F3 to 4 (AUC: 0.879 vs SUV [*P* = .008] vs CEI [*P* = .023]), and F0 to 3 and F4 (AUC: 0.815 vs SUV [*P* = .1388] vs CEI [*P* = .014]) stages, combined SUV and CEI showed higher AUCs than SUV or CEI alone. When comparing the relatively fibrosis-free and advanced-fibrosis groups (stages F0 to 2 and F3 to 4, respectively), the combination of SUV and CEI showed significantly higher AUC values than SUV or CEI alone.

## 4. Discussion

Asialoglycoprotein receptors are present on the surface of a normal mammalian liver.^[37,38]^ Although a decrease in the number of receptors is observed in patients with hepatic impairment, ^99m^Tc-GSA is used to assess hepatic reserve, as the number of asialoglycoprotein receptors present on hepatocytes correlates well with hepatocyte function.^[39–41]^ ^99m^Tc-GSA is a radiopharmaceutical that binds specifically to the asialoglycoprotein receptor and is less likely to be affected by extrahepatic factors.^[13]^ Recently, the combination of SPECT and CT in ^99m^Tc-GSA scintigraphy has been used to assess various liver diseases, as it allows the local assessment of hepatocyte function and radioisotope quantification.^[29,39,42–44]^ In this study, SUV was used as an indicator of liver fibrosis, and it is reproducible and easy to calculate. The calculation of SUV by SPECT-CT, similar to positron emission tomography, and may be affected by partial volume effects, depending on the size of the object being measured. However, the liver is the largest organ in the body and should be less affected by partial volume effects.^[45]^ In the assessment of liver fibrosis using SUV with SPECT-CT fusion imaging, it is possible to set ROIs on CT to assess the whole liver and individual regions by measuring resected and residual liver volumes.^[42]^

Gd-EOB-DTPA is a liver-specific MRI contrast agent, and the indices obtained from contrast images assess both liver function and fibrosis.^[23,46,47]^ The significant decrease in the contrast index with advanced-fibrosis compared with mild-fibrosis is due to the reduced uptake of disodium gadoxetate by hepatocytes as a result of reduced normal hepatocyte numbers caused by fibrotic tissue growth or severe hepatocellular dysfunction, degeneration, and necrosis.^[48,49]^ The accumulation of Gd-EOB-DTPA in the liver is associated with several other factors, including individual indocyanine green clearance and plasma bilirubin levels.^[26]^ Several methods for assessing liver fibrosis using EOB have been reported.^[50–52]^ Among all the parameters mentioned above, CEI is the only parameter that has been comprehensively assessed in several studies and is considered relatively common.^[53]^ Previous studies have also shown that CEI is useful for determining regional and total liver reserves and can be similarly used to assess fibrosis.^[54,55]^

To the best of our knowledge, our study is the first to use ^99m^Tc-GSA scintigraphy and Gd-EOB-DTPA-enhanced MRI as a multimodality approach for liver fibrosis staging. A variety of liver function tests are currently available, most of these tests provide an overall assessment of liver function. However, to determine the extent of hepatic resection, it is important to assess the local background liver that should be preserved. Both ^99m^Tc-GSA scintigraphy and Gd-EOB-DTPA-enhanced MRI scans allow for localized assessment by ROI and may help hepatosurgeons to establish the extent more accurately. Moreover, combining measurements with different accumulation mechanisms enables a more robust assessment. MRI has a higher spatial resolution than ^99m^Tc-GSA scintigraphy, which allows for more accurate localization, but in this study, no significant differences were found between F3 and F4. The inclusion of ^99m^Tc-GSA scintigraphy, which shows a significant difference between F3 and F4 in the assessment may allow a more accurate assessment of background liver. In addition, assessment of fibrosis through imaging can provide additional information such as anatomical images for morphological analysis, levels of liver function, and assessment of localized lesions in the liver.

Various methods for noninvasive assessment of liver fibrosis have been proposed and used in clinical practice. Combining the 2 tests allows for a more robust assessment, but at an increased cost. It is not cost realistic to perform MRI and ^99m^Tc-GSA scintigraphy for the sole purpose of assessing liver fibrosis. However, if these methods are used in preoperative liver resection examination, as well as US and blood tests, for other purposes than the assessment of liver fibrosis, such as the assessment of liver function and lesion progression, the method shown in this study can be used to assess the background liver at no additional cost. In the future, it is necessary to evaluate whether ^99m^Tc-GSA scintigraphy and MRI, which allow imaging evaluation, can be combined with other modalities performed preoperatively to obtain more detailed preoperative background liver information using the data already obtained.

The SUV decreased significantly with increasing fibrosis; however, no difference was observed among F0, F1, and F2, which indicated no or relatively mild-fibrosis. This is consistent with the finding that, the GSA test, when performed to estimate liver function, is not remarkably sensitive in differentiating mild liver damage from normal liver.^[56,57]^ In contrast, CEI showed significant differences between F0 and F2 but no significant differences between F3 and F4. This is similar to the results of previous studies, suggesting that discrimination may be inadequate in livers with relatively high levels of fibrosis.^[26]^ In the ROC analysis, the combination of SUV and CEI showed the highest AUC in both fibrosis groups. In particular, in the comparison of the fibrosis group with the no-fibrosis or mild-fibrosis groups, SUV + CEI showed significantly higher AUCs than SUV or CEI alone in discriminating between fibrosis and no or mild-fibrosis, suggesting that the combination of SUV + CEI has greater diagnostic performance than the individual indices. Therefore, the combination of the 2 indices may provide a more accurate diagnosis.

Our study has some limitations. First, as the study was retrospective, case selection bias was unavoidable, which could be due to the heterogeneity of the etiology of liver disease in the included patients. Second, the study was a single-center evaluation and did not consider the influence of GSA and MRI imaging conditions on the results. In addition, due to the study retrospective nature, the level of evidence was lower. Further prospective validation in larger populations is needed for the assessment of liver fibrosis. Finally, The uptake of Gd-EOB-DTPA is affected by inflammatory responses, and the observed reduction in uptake may not be entirely due to fibrosis.^[58]^

## 5. Conclusions

Combining SUV and CEI provides high accuracy for grading liver fibrosis, especially in differentiating between F0 and 2, F3 and 4. SUV and Gd-EOB-DTPA-enhanced MRI can be used as noninvasive diagnostic methods to guide the selection of clinical treatment options for patients with liver diseases.

## Acknowledgments

We are grateful to Yukinori Okada, MD, PhD (St. Marianna University School of Medicine) for her assistance with the statistical analyses. I would also like to take this opportunity to thank Shigeki Kuzuhara, MD, PhD for years of collaboration and advice.

## Author contributions

**Conceptualization:** Ryotaro Tokorodani

**Data curation:** Toshiaki Kume, Naoya Hayashi

**Project administration:** Ryotaro Tokorodani, Takuji Yamagami

**Validation:** Hiromitu Daisaki

**Writing—original draft:** Ryotaro Tokorodani

**Supervision:** Takuji Yamagami, Hitomi Iwasa

## References

[1] Farges O Malassagne B Flejou JF. Risk of major liver resection in patients with underlying chronic liver disease: a reappraisal. Ann Surg. 1999;229:210–5. 10024102 10.1097/00000658-199902000-00008 PMC1191633

[2] Nagino M Kamiya J Nishio H. Two hundred forty consecutive portal vein embolizations before extended hepatectomy for biliary cancer: surgical outcome and long-term follow-up. Ann Surg. 2006;243:364–72. 16495702 10.1097/01.sla.0000201482.11876.14 PMC1448943

[3] Zarzavadjian Le Bian A Costi R Sbai-Idrissi MS. Liver resection and metabolic disorders: an undescribed mechanism leading to postoperative mortality. World J Gastroenterol. 2014;20:14455–62. 25339832 10.3748/wjg.v20.i39.14455 PMC4202374

[4] Wang Q Fiel MI Blank S. Impact of liver fibrosis on prognosis following liver resection for hepatitis B-associated hepatocellular carcinoma. Br J Cancer. 2013;109:573–81. 23846171 10.1038/bjc.2013.352 PMC3738114

[5] Rockey DC Caldwell SH Goodman ZD. American Association for the Study of Liver Diseases. Liver biopsy. Hepatology. 2009;49:1017–44. 19243014 10.1002/hep.22742

[6] Ratziu V Charlotte F Heurtier A. Sampling variability of liver biopsy in nonalcoholic fatty liver disease. Gastroenterology. 2005;128:1898–906. 15940625 10.1053/j.gastro.2005.03.084

[7] Chin JL Pavlides M Moolla A. Non-invasive markers of liver fibrosis: adjuncts or alternatives to liver biopsy? Front Pharmacol. 2016;7:159. 27378924 10.3389/fphar.2016.00159 PMC4913110

[8] Talwalkar JA Yin M Fidler JL. Magnetic resonance imaging of hepatic fibrosis: emerging clinical applications. Hepatology. 2008;47:332–42. 18161879 10.1002/hep.21972

[9] Verloh N Einspieler I Utpatel K. In vivo confirmation of altered hepatic glucose metabolism in patients with liver fibrosis/cirrhosis by 18F-FDG PET/CT. EJNMMI Res. 2018;8:98. 30414009 10.1186/s13550-018-0452-y PMC6226405

[10] Yang L Ding Y Rao S. Staging liver fibrosis in chronic hepatitis B with T1 relaxation time index on gadoxetic acid-enhanced MRI: comparison with aspartate aminotransferase-to-platelet ratio index and FIB-4. J Magn Reson Imaging. 2017;45:1186–94. 27563840 10.1002/jmri.25440

[11] Pan S Wang L Xin J. Combining 18F-FDG PET and Gd-EOB-DTPA-enhanced MRI for staging liver fibrosis. Life Sci. 2021;269:119086. 33476634 10.1016/j.lfs.2021.119086

[12] Zheng J Guo H Zeng J. Two-dimensional Shear-Wave elastography and conventional US: the optimal evaluation of liver fibrosis and cirrhosis. Radiology. 2015;275:290–300. 25575116 10.1148/radiol.14140828

[13] Kotani K Kawabe J Higashiyama S. Heterogeneous liver uptake of Tc-99m-GSA as quantified through SPECT/CT helps to evaluate the degree of liver fibrosis: A retrospective observational study. Med (Baltimore). 2018;97:e11765. 10.1097/MD.0000000000011765 PMC6081161 30075603

[14] Tsochatzis EA Gurusamy KS Ntaoula S. Elastography for the diagnosis of severity of fibrosis in chronic liver disease: a meta-analysis of diagnostic accuracy. J Hepatol. 2011;54:650–9. 21146892 10.1016/j.jhep.2010.07.033

[15] Friedrich-Rust M Ong MF Martens S. Performance of transient elastography for the staging of liver fibrosis: a meta-analysis. Gastroenterology. 2008;134:960–74. 18395077 10.1053/j.gastro.2008.01.034

[16] Virarkar M Morani AC Taggart MW. Liver fibrosis assessment. Semin Ultrasound CT MR. 2021;42:381–9. 34130850 10.1053/j.sult.2021.03.003

[17] Hudert CA Tzschätzsch H Guo J. US time-harmonic elastography: detection of liver fibrosis in adolescents with extreme obesity with nonalcoholic fatty liver disease. Radiology. 2018;288:99–106. 29762096 10.1148/radiol.2018172928

[18] Millonig G Friedrich S Adolf S. Liver stiffness is directly influenced by central venous pressure. J Hepatol. 2010;52:206–10. 20022130 10.1016/j.jhep.2009.11.018

[19] Song ZZ. Extrahepatic cholestasis and liver stiffness by transient elastography. Hepatology. 2009;49:1053–1053. 10.1002/hep.22804 19241481

[20] Cosgrove D Piscaglia F Bamber J. EFSUMB guidelines and recommendations on the clinical use of ultrasound elastography. Part 2: Clinical applications. Ultraschall Med. 2013;34:238–53. 23605169 10.1055/s-0033-1335375

[21] Hines CDG Bley TA Lindstrom MJ. Repeatability of magnetic resonance elastography for quantification of hepatic stiffness. J Magn Reson Imaging. 2010;31:725–31. 20187219 10.1002/jmri.22066 PMC2901399

[22] Motosugi U Ichikawa T Sano K. Magnetic resonance elastography of the liver: preliminary results and estimation of inter-rater reliability. Jpn J Radiol. 2010;28:623–7. 20972864 10.1007/s11604-010-0478-1 PMC4584141

[23] Palmucci S. Focal liver lesions detection and characterization: the advantages of gadoxetic acid-enhanced liver MRI. World J Hepatol. 2014;6:477–85. 25067999 10.4254/wjh.v6.i7.477 PMC4110539

[24] Fayad LM Kamel IR Mitchell DG. Functional MR cholangiography: diagnosis of functional abnormalities of the gallbladder and biliary tree. AJR Am J Roentgenol. 2005;184:1563–71. 15855116 10.2214/ajr.184.5.01841563

[25] Golfieri R Grazioli L Orlando E. Which is the best MRI marker of malignancy for atypical cirrhotic nodules: hypointensity in hepatobiliary phase alone or combined with other features? Classification after Gd-EOB-DTPA administration. J Magn Reson Imaging. 2012;36:648–57. 22592930 10.1002/jmri.23685

[26] Watanabe H Kanematsu M Goshima S. Staging hepatic fibrosis: comparison of gadoxetate disodium-enhanced and diffusion-weighted MR imaging–preliminary observations. Radiology. 2011;259:142–50. 21248234 10.1148/radiol.10100621

[27] Juluru K Talal AH Yantiss RK. Diagnostic accuracy of intracellular uptake rates calculated using dynamic Gd-EOB-DTPA–enhanced MRI for hepatic fibrosis stage. J Magn Reson Imaging. 2017;45:1177–85. 27527820 10.1002/jmri.25431 PMC5313385

[28] Tokorodani R Kume T Daikoku K. [Evaluation of the validity of ROI setting in CEI used for the assessment of liver] [article in Japanese]. Nihon Hoshasen Gijutsu Gakkai Zasshi. 2022;78:44–52. 35046221 10.6009/jjrt.780105

[29] Tokorodani R Sumiyoshi T Okabayashi T. Liver fibrosis assessment using 99mTc-GSA SPECT/CT fusion imaging. Jpn J Radiol. 2019;37:315–20. 30656542 10.1007/s11604-019-00810-w

[30] Taniguchi M Okizaki A Watanabe K. Hepatic clearance measured with (99m)Tc-GSA single-photon emission computed tomography to estimate liver fibrosis. World J Gastroenterol. 2014;20:16714–20. 25469042 10.3748/wjg.v20.i44.16714 PMC4248217

[31] Iguchi T Sato S Kouno Y. Comparison of Tc-99m-GSA scintigraphy with hepatic fibrosis and regeneration in patients with hepatectomy. Ann Nucl Med. 2003;17:227–33. 12846545 10.1007/BF02990026

[32] Suh MS Lee WW Kim YK. Maximum standardized uptake value of (99m)Tc hydroxymethylene diphosphonate SPECT/CT for the evaluation of temporomandibular joint disorder. Radiology. 2016;280:890–6. 27035060 10.1148/radiol.2016152294

[33] Kinahan PE Fletcher JW. Positron emission tomography-computed tomography standardized uptake values in clinical practice and assessing response to therapy. Semin Ultrasound CT MR. 2010;31:496–505. 21147377 10.1053/j.sult.2010.10.001 PMC3026294

[34] Oshiro Y Ohkohchi N. Three-dimensional liver surgery simulation: computer-assisted surgical planning with three-dimensional simulation software and three-dimensional printing. Tissue Eng Part A. 2017;23:474–80. 28343411 10.1089/ten.TEA.2016.0528

[35] Ludwig J. The nomenclature of chronic active hepatitis: an obituary. Gastroenterology. 1993;105:274–8. 8514045 10.1016/0016-5085(93)90037-d

[36] DeLong ER DeLong DM Clarke-Pearson DL. Comparing the areas under two or more correlated receiver operating characteristic curves: a nonparametric approach. Biometrics. 1988;44:837–45. 3203132

[37] Ashwell G Morell AG. The role of surface carbohydrates in the hepatic recognition and transport of circulating glycoproteins. Adv Enzymol Relat Areas Mol Biol. 1974;41:99–128. 4609051 10.1002/9780470122860.ch3

[38] Sawamura T Kawasato S Shiozaki Y. Decrease of a hepatic binding protein specific for asialoglycoproteins with accumulation of serum asialoglycoproteins in galactosamine-treated rats. Gastroenterology. 1981;81:527–33. 7250640

[39] Okabayashi T Shima Y Morita S. Liver function assessment using technetium 99m-galactosyl single-photon emission computed tomography/CT fusion imaging: a prospective trial. J Am Coll Surg. 2017;225:789–97. 28912030 10.1016/j.jamcollsurg.2017.08.021

[40] Akaki S Mitsumori A Kanazawa S. Technetium-99m-DTPA-galactosyl human serum albumin liver scintigraphy evaluation of regional CT/MRI attenuation/signal intensity differences. J Nucl Med. 1998;39:529–32. 9529304

[41] Matsuzaki S Onda M Tajiri T. Hepatic lobar differences in progression of chronic liver disease: correlation of asialoglycoprotein scintigraphy and hepatic functional reserve. Hepatology. 1997;25:828–32. 9096583 10.1002/hep.510250407

[42] Sumiyoshi T Shima Y Okabayashi T. Liver function assessment using 99mTc-GSA single-photon emission computed tomography (SPECT)/CT fusion imaging in hilar bile duct cancer: a retrospective study. Surgery. 2016;160:118–26. 27059635 10.1016/j.surg.2016.02.009

[43] Iimuro Y Kashiwagi T Yamanaka J. Preoperative estimation of asialoglycoprotein receptor expression in the remnant liver from CT/99mTc-GSA SPECT fusion images correlates well with postoperative liver function parameters. J Hepato Pancreat Sci. 2010;17:673–81. 10.1007/s00534-010-0264-6 20703846

[44] Yoshida M Shiraishi S Sakaguchi F. Fused 99m-Tc-GSA SPECT/CT imaging for the preoperative evaluation of postoperative liver function: can the liver uptake index predict postoperative hepatic functional reserve? Jpn J Radiol. 2012;30:255–62. 22302293 10.1007/s11604-011-0041-8

[45] Soret M Bacharach SL Buvat I. Partial-volume effect in PET tumor imaging. J Nucl Med. 2007;48:932–45. 17504879 10.2967/jnumed.106.035774

[46] Schuhmann-Giampieri G Schmitt-Willich H Press WR. Preclinical evaluation of Gd-EOB-DTPA as a contrast agent in MR imaging of the hepatobiliary system. Radiology. 1992;183:59–64. 1549695 10.1148/radiology.183.1.1549695

[47] Weinmann HJ Schuhmann-Giampieri G Schmitt-Willich H. A new lipophilic gadolinium chelate as a tissue-specific contrast medium for MRI. Magn Reson Med. 1991;22:233–7. 1812351 10.1002/mrm.1910220214

[48] Besa C Bane O Jajamovich G. 3D T1 relaxometry pre and post gadoxetic acid injection for the assessment of liver cirrhosis and liver function. Magn Reson Imaging. 2015;33:1075–82. 26119422 10.1016/j.mri.2015.06.013

[49] Sheng RF Wang HQ Yang L. Assessment of liver fibrosis using T1 mapping on Gd-EOB-DTPA-enhanced magnetic resonance. Dig Liver Dis. 2017;49:789–95. 28237298 10.1016/j.dld.2017.02.006

[50] Shimizu J Dono K Gotoh M. Evaluation of regional liver function by gadolinium-EOB-DTPA-enhanced MR imaging. Dig Dis Sci. 1999;44:1330–7. 10489914 10.1023/a:1026679113772

[51] Motosugi U Ichikawa T Muhi A. Magnetic resonance elastography as a predictor of insufficient liver enhancement on gadoxetic acid-enhanced hepatocyte-phase magnetic resonance imaging in patients with Type C hepatitis and Child-Pugh Class A disease. Invest Radiol. 2012;47:566–70. 22955025 10.1097/RLI.0b013e318260ac9e

[52] Kanki A Tamada T Higaki A. Hepatic parenchymal enhancement at Gd-EOB-DTPA-enhanced MR imaging: correlation with morphological grading of severity in cirrhosis and chronic hepatitis. Magn Reson Imaging. 2012;30:356–60. 22227353 10.1016/j.mri.2011.11.002

[53] Tajima T Takao H Akai H. Relationship between liver function and liver signal intensity in hepatobiliary phase of gadolinium ethoxybenzyl diethylenetriamine pentaacetic acid-enhanced magnetic resonance imaging. J Comput Assist Tomogr. 2010;34:362–6. 20498536 10.1097/RCT.0b013e3181cd3304

[54] Bae KE Kim SY Lee SS. Assessment of hepatic function with Gd-EOB-DTPA-enhanced hepatic MRI. Dig Dis. 2012;30:617–22. 23258104 10.1159/000343092

[55] Li X Liu H Wang R. Gadoxetate-disodium-enhanced magnetic resonance imaging for liver fibrosis staging: a systematic review and meta-analysis. Clin Radiol. 2020;75:319.e11–9. 10.1016/j.crad.2019.11.001 31831141

[56] De Groote J Desmet VJ Gedigk P. A classification of chronic hepatitis. Lancet. 1968;292:626–8. 10.1016/s0140-6736(68)90710-1 4175170

[57] Hata S Ishii K. Effect of galactose on binding and endocytosis of asialoglycoprotein in cultured rat hepatocytes. Ann Nucl Med. 1998;12:255–9. 9839486 10.1007/BF03164910

[58] Verloh N Probst U Utpatel K. Influence of hepatic fibrosis and inflammation: correlation between histopathological changes and Gd-EOB-DTPA-enhanced MR imaging. PLoS One. 2019;14:e0215752. 31083680 10.1371/journal.pone.0215752 PMC6513096

